# Ultrahigh affinity Raman probe for targeted live cell imaging of prostate cancer†
†Electronic supplementary information (ESI) available: Fig. S1–S7. See DOI: 10.1039/c6sc01739h



**DOI:** 10.1039/c6sc01739h

**Published:** 2016-07-15

**Authors:** Ming Li, Sangeeta Ray Banerjee, Chao Zheng, Martin G. Pomper, Ishan Barman

**Affiliations:** a Department of Mechanical Engineering , Johns Hopkins University , Baltimore , Maryland 21218 , USA . Email: liming0823@gmail.com ; Email: ibarman@jhu.edu; b The Sidney Kimmel Comprehensive Cancer Center , Johns Hopkins University School of Medicine , Baltimore , Maryland 21287 , USA . Email: mpomper@jhmi.edu; c The Russell H. Morgan Department of Radiology and Radiological Sciences , Johns Hopkins University School of Medicine , Baltimore , Maryland 21287 , USA; d Department of Breast Surgery , The Second Hospital of Shandong University , Jinan , Shandong 25000 , China

## Abstract

Precise visualization of tumor margins with characterization of microscopic tumor invasion are unmet needs in prostate oncology that demand approaches with high sensitivity and specificity. To address those needs we report surface-enhanced Raman scattering (SERS) based optical imaging for prostate cancer using a combination of live cell Raman microscopy, optimally engineered SERS tags and a urea-based small-molecule inhibitor of prostate-specific membrane antigen (PSMA) as a targeting moiety. We develop gold nanostar based SERS agents that offer ultrahigh binding affinity to PSMA with nearly four orders of magnitude lower IC_50_ values in relation to existing clinical imaging agents. This combination enables selective recognition of prostate cancer cells, and facilitates quantitative and photostable Raman measurements. Using Raman microscopy to analyze phenotypically similar prostate cancer cell lines differing only in PSMA expression, we demonstrate facile, site-selective recognition using as low as 20 pM of the SERS agent for imaging, opening the door for spectroscopic detection of prostate and other PSMA-expressing tumors *in vivo*.

## Abstract 2

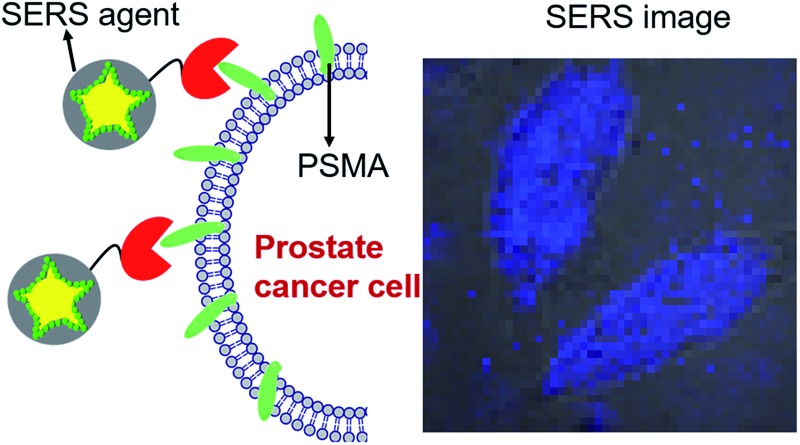
Leveraging optimally engineered SERS tags and urea-based small-molecule inhibitor of PSMA, we report an ultrahigh binding affinity imaging nanoplex for castrate resistant prostate cancer and demonstrate live single cell vibrational spectroscopic imaging at ultralow concentrations.

## Introduction

The past decade has witnessed considerable effort in the development of platforms for rapid screening, theranostics, and targeted therapy of prostate cancer.^1–3^ Despite those endeavors, prostate cancer remains the second leading cause of cancer death in American men. The American Cancer Society estimates 220 800 new cases and 27 540 deaths attributable to prostate cancer in 2015 in the US alone.^4^ Two challenges contribute to morbidity and mortality from prostate cancer. First, the inability to separate tumors that will progress to life-threatening disease from those that will remain indolent confounds precision therapy. Second, the difficulty in assuring a clear surgical margin during prostatectomy often leads to local recurrence that may progress to metastasis.^5^ Since existing modalities do not provide reliable information on the true extent of tumors, a significant number of patients who undergo prostatectomy are subjected to (unnecessary) adjuvant radiation therapy with its attendant morbidity and expense.^6^

In this milieu, new imaging strategies for non-invasive, real-time monitoring of molecular changes associated with prostate cancer are desirable. Such molecular imaging can be used to map microscopic satellite lesions, to differentiate aggressive subtypes, and to delineate tumor margins precisely during resection. Fluorescence imaging has been proposed for image-guided surgery in light of the emergence of sensitive molecular probes, especially in the tissue-transparent near infrared (NIR) window.^7^ Yet the presence of tissue autofluorescence and photobleaching of fluorophore emission hinders specific and quantitative measurements.

Owing to its high molecular specificity, Raman spectroscopy has surfaced as an attractive candidate to illustrate complex, spatially localized molecular interactions in cells and tissue. It relies on the inelastic scattering of photons upon interaction with molecular vibrations of the specimen and offers a quantitative, real-time measurement of molecular composition. Studies by us and others have sought to use such spectral markers as new routes to recognition of cell types within tissues as well as objective cancer detection.^8,9^ Nevertheless, the inherently weak Raman signal limits its utility for extensive cellular and tissue imaging. Surface-enhanced Raman scattering (SERS) exploits the phenomenon of localized surface plasmon resonance (LSPR) to overcome that limitation and has emerged as a potent analytical tool by virtue of its near single-molecule sensitivity of detection and fingerprinting capability.^10–12^ The signal enhancement (*ca.* 10^6^ to 10^12^) principally arises from the proximity of the Raman reporter to the intensely localized plasmonic fields of structured metallic nanoparticles.^13^

Those salient features, coupled with the photostability of Raman signals and lower matrix interference, provide a unique opportunity for targeted molecular imaging *via* sensing of biomarkers characteristic of cancer progression.^14,15^ SERS measurements of prostate tissue chemistry, however, are largely unexplored and there is a lack of viable agents that can transduce the differential presence of prostate cancer markers at the cellular level to measurable signals. Herein we report a SERS based imaging approach to visualize castration resistant prostate cancer cells using a combination of Raman spectroscopic imaging, SERS tags and a urea-based small-molecule inhibitor of prostate-specific membrane antigen (PSMA).

For the SERS imaging platform we first optimized nanoprobe signals through designed resonance conditions of the nanoparticle, which was a gold nanostar (GNS). We previously investigated the shape effect of gold nanoparticles, and found that GNSs offer a superior SERS substrate with extremely high sensitivity.^16^ GNSs present self-generated “hot-spots” that result in substantive signal enhancement without the need to aggregate nanoparticles.^17–19^ Second, we hypothesized that PSMA, a type II integral membrane protein that is significantly over-expressed on the cell surface of most prostate cancers but particularly in castration-resistant, advanced and metastatic disease,^20,21^ could serve as a relevant target for SERS based imaging. The choice of the target was dictated by the extracellular location of the ligand binding site and the high receptor concentration per cell (*ca.* 3.2 μM per cell volume).^22^ Finally, we employed Raman spectroscopic imaging to analyze the binding and uptake of the SERS agent as well as its brightness and sub-cellular signal localization in prostate tumor cells.

## Results and discussion

**Fig. 1:**
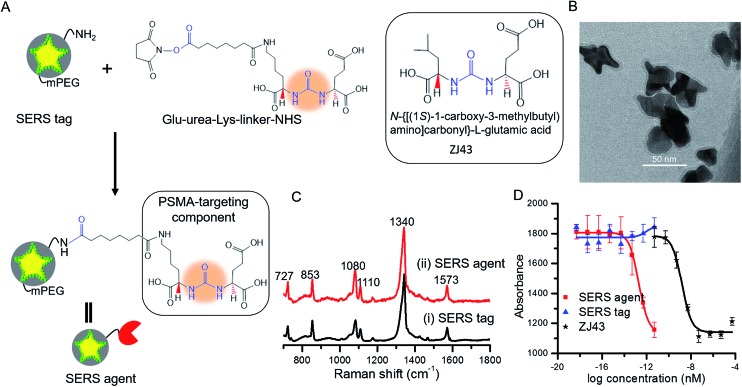
(A) Schematic of SERS agent following conjugation with the urea based PSMA targeting component. Glu-urea-Lys-linker-NHS was grafted onto the SERS tag surface through amine coupling chemistry to prepare the final SERS agent. The molecular structure of the known PSMA inhibitor *N*-{[(1*S*)-1-carboxy-3-methylbutyl) amino]carbonyl}-l-glutamic acid (ZJ43) is also shown.^5^ (B) TEM image of the sandwich SERS tag encoded with 4-nitrothiophenol (NTP). (C) SERS spectra of (i) the SERS tag and (ii) SERS agent exhibiting correspondence with the signature of the Raman reporter molecule. (D) IC_50_ curves of SERS tag, SERS agent and ZJ43.

The SERS tags were prepared by sandwiching a layer of Raman reporter molecule, 4-nitrothiophenol (NTP), between the GNS and the thin silica protective layer (Fig. 1A).^16,23^ By modulating the protrusion length, density and core size, we have recently established the tunable plasmonic properties of the GNS.^24^ Here, we used the GNS with LSPR absorbance maximum of 750 nm as the plasmonic core for the SERS tag (Fig. S2†). The binding of NTP onto the GNS surface through strong S–Au interactions led to a single layer of NTP, and the silica protective layer prevented the leakage of NTP into the surrounding medium, stabilizing the SERS signal (Fig. S3†). The synthesized SERS tags exhibited high brightness, stemming from electric field concentration around GNS tips and the encapsulation of a large number of NTP molecules in a single tag. Although previous work showed that silica exerts little *in vivo* cytotoxicity,^25^ we further modified the SERS tag with mPEG–silane to alleviate any residual biocompatibility concerns.^26^ Characterization *via* extinction measurements showed a 30 nm red-shift in LSPR of SERS tag compared with the bare GNS (Fig. S2†), which is attributed to the change in refractive index after silica coating. TEM images confirmed the successful coating of GNS with a 3–4 nm silica layer (Fig. 1B and S1†).

For functionalization of the SERS tag, we selected urea based small molecules possessing high affinity and specificity to PSMA, analogs of which have been successfully deployed for PET, SPECT and fluorescence imaging.^27–29^ The Glu-urea-Lys-linker-NHS was grafted onto the surface of the SERS tag through amine coupling chemistry (Fig. 1A). The functional urea component can specifically recognize zinc within the active site of PSMA.^5,20^ A full monolayer of urea based linker coverage (under a 1 : 2 molar ratio of APTMS : mPEG–silane and complete reaction of APTMS molecules with linkers) would imply approximately 3300 linkers per SERS agent, since the aforementioned moieties adsorb to the SERS tag surface with a molecular footprint of *ca.* 50 Å^2^.^30,31^ The experimental loadings could, though, be lower as reported recently for a nanorod-derived SERS agent.^32^

Fig. 1C shows spectra acquired from the SERS tag and SERS agent modified with the PSMA targeting moiety. Several intense Raman features characteristic of NTP are observed,^24,33^ including bands at 727 cm^–1^ (wagging vibrations of C–H, C–S and C–C), 853 cm^–1^ (wagging vibration of C–H), 1080 cm^–1^ (stretching vibration of C–S), 1110 cm^–1^ (bending vibration of C–H), 1340 cm^–1^ (stretching vibration of N–O), and 1573 cm^–1^ (stretching vibration of phenyl ring). In relation to the photostability of the SERS agents, we note that no laser-induced photoreaction (such as dimerization) of NTP was observed, consistent with previous studies that show gold is an inefficient catalyst for the dimerization of NTP when exposed to a 785 nm near-infrared laser.^34–36^

Next, the PSMA inhibitory activity of the intact SERS agent was examined using an established fluorescence-based assay.^37^ Lysates of PSMA+ PC3 PIP cell extracts were incubated with the agent in the presence of *N*-acetyl-aspartyl-glutamate and the amount of reduced glutamate was determined using a fluorescence microplate reader after incubating with Amplex® Red. The unmodified SERS tag and the small-molecule PSMA inhibitor (ZJ43) were used as controls in the study (Fig. 1D).^5^ The SERS agent exhibited surprisingly high affinity toward PSMA+ PC3 PIP cells with an IC_50_ value of 2.0 × 10^–13^ nM, nearly four orders of magnitude lower than the value of ZJ43 alone (1.6 × 10^–9^ nM). Such high affinity suggests better long-term stability for SERS imaging, and also improved pharmacokinetics and prolonged contact time to the targeted sites. The use of the small-molecule inhibitor for construction of the final SERS agent, as opposed to antibodies or aptamers, renders substantive advantages in terms of formulation stability as well as synthesis purity, efficiency and economy.^38^

**Fig. 2:**
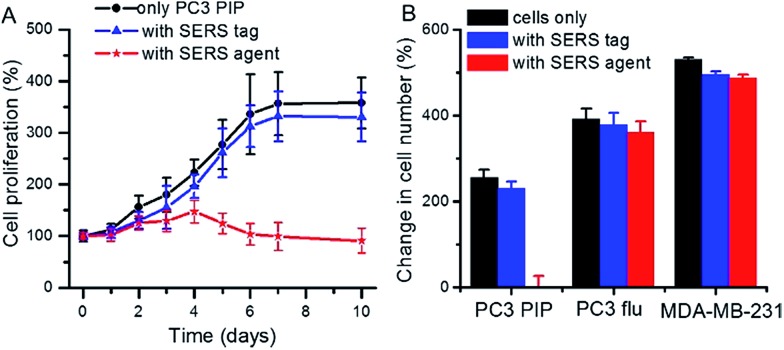
(A) Cell proliferation of PSMA+ PC3 PIP cells in the presence or absence of SERS tag and SERS agent (50 pM). (B) Change of cell number of PSMA+ PC3 PIP, PSMA– PC3 flu and breast cancer MDA-MB-231 cells in the absence or presence of SERS tag and SERS agent after incubation for 7 days.

We also investigated the effect of the SERS tag and the targeted SERS agent on cell proliferation in PSMA+ PC3 PIP cells. In determining binding specificity, cellular cytotoxicity and/or tumor uptake, we prefer comparing isogenic PSMA+ PC3 PIP to PSMA – PC3 flu cells as the two human cell lines are identical, differing only in PSMA expression.^3,5^ Fig. 2A displays the observed growth of cells in the presence and absence of SERS tags and SERS agents, respectively. The PSMA+ PC3 PIP cells proliferated over the prolonged incubation time and followed the same growth trend in both absence and presence of the SERS tags, but reached saturation after a period of 6 days. In contrast, growth of the PSMA+ PC3 PIP cells decreased in the presence of the targeted SERS agent, and the number of cells even showed a slight decrease after incubation for 4 days. The sizable differences in our observations reflect the capacity of the SERS agent to inhibit cell proliferation in PSMA+ cells. Furthermore, to examine the specificity of cell kill *in vitro*, PSMA– PC3 flu and MDA-MB-231 cells were used as controls. As can be seen from Fig. 2B, the MDA-MB-231 cell grew faster in comparison with PSMA+ PC3 PIP and PSMA– PC3 flu cells, while PSMA+ PC3 PIP cells demonstrated the slowest growth rate. That is consistent with the fact that MDA-MB-231 cells grow faster than PSMA+ PC3 PIP and PSMA– PC3 flu cells under standard conditions, without addition of any SERS tag or SERS agent. Critically, the SERS tag and SERS agent have minimal impact on the growth of both PSMA– PC3 flu and MDA-MB-231 cells – in sharp contrast to the observed impact of the SERS agent on the PSMA+ PC3 PIP cells. This also confirms that the surface modification moieties of SERS agents APTMS and mPEG have insignificant inhibitory activity. Collectively, these findings highlight the specificity of the developed SERS agents for PSMA+ PC3 PIP cells, setting the foundation for quantitative Raman measurements of this key biomarker and its subcellular localization in specific cell types.

**Fig. 3:**
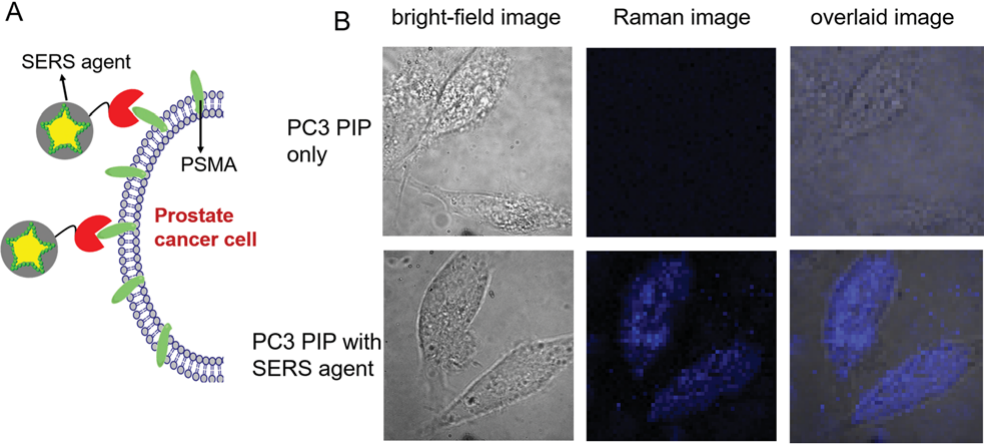
(A) Schematic illustration of the targeting of live cells by SERS agents. (B) Bright-field and spatially mapped 1340 cm^–1^ Raman peak intensity images of PSMA+ PC3 PIP cells in the absence/presence of SERS agent (50 pM). The SERS images were constructed on the basis of the integrated intensity of the 1340 cm^–1^ Raman band.

To evaluate the targeted imaging of live cells, we first performed proof-of-concept Raman measurements in PSMA+ PC3 PIP cells, separately in the absence and presence of SERS agents (Fig. 3). Fig. 3A recapitulates the principle of operation of targeted SERS imaging employed in this study. Briefly, a home-built inverted confocal Raman microscope, similar in design to that described in our recent publication,^33^ was used for acquisition of the time-lapsed hyperspectral datasets (*x* – *y* – *λ*). The use of galvano mirrors in this design, rather than mechanical stage movement, to facilitate cellular mapping substantially enhances the acquisition speed and reduces motion-induced image artifacts. A NIR laser beam (*λ*_ex_ = 785 nm) was focused onto a single cell and it was scanned with 50 × 50 pixels over an 80 μm × 80 μm area. Customized quartz-bottomed Petri dishes were used for culturing the cells in order to minimize spectral interference from the substrate during live cell imaging. Expectedly, in the absence of the SERS agent, no significant Raman features could be discerned from the hyperspectral data, which principally consisted of broad autofluorescence emission. However, after PSMA+ PC3 PIP cells were incubated in 50 pM SERS agent, intense Raman signatures, characteristic of the NTP modes, were observed. In fact, the intense brightness of the SERS agents allowed us to operate with a low laser power of 5 mW, which coupled with the NIR wavelength ensured that the cells remain viable during and following the course of the measurements. We constructed the SERS image of examined cells from the spatially mapped intensity of the stretching vibration of N–O at 1340 cm^–1^ after background subtraction (Fig. 3B). The live PSMA+ PC3 PIP cells can be clearly demarcated from the SERS image, which shows a direct correspondence with the bright-field images. The SERS image also verifies that very few of the agents were non-specifically distributed through the medium. Our measurements confirm the rich molecular detail and photostability of the vibrational spectroscopic approach, particularly when contrasted with fluorescence imaging.

**Fig. 4:**
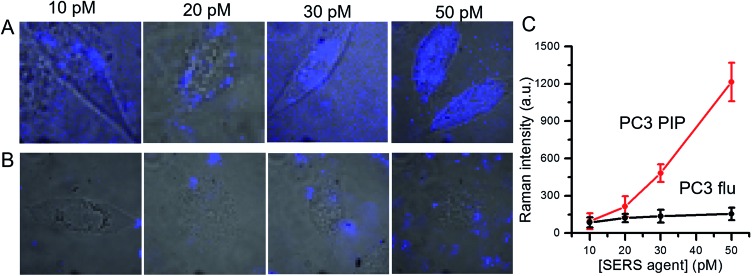
Dose-dependent SERS images of (A) PSMA+ PC3 PIP and (B) PSMA– PC3 flu cells following incubation in various concentrations (10, 20, 30 and 50 pM) of SERS agents. The SERS images were overlaid with bright-field images to aid understanding of the spatial localization of the agents. (C) Concentration-dependent SERS intensity determined by averaging the SERS intensity over the pixels on a single cell. The SERS images were constructed on the basis of the integrated intensity of the 1340 cm^–1^ Raman band.

We further examined dose-dependent SERS imaging in PSMA+ PC3 PIP and PSMA– PC3 flu cells (Fig. 4A, B and S5†). With increasing SERS agent concentration from 10 pM to 50 pM, the intensity of the 1340 cm^–1^ NTP peak showed a corresponding rise for the PSMA+ PC3 PIP cells. The SERS intensity did not exhibit evident trends for the PSMA– PC3 flu cells with increasing SERS agent concentration. To quantify these observations, we computed the average SERS intensity per cell by dividing the aggregate SERS intensity by the number of pixels in a single cell (Fig. 4C). The SERS intensity shows a near linear increase with increasing concentration of the SERS agent for the PSMA+ PC3 PIP cells, especially at higher concentrations; on the other hand, no statistically significant differences are noted for the PSMA– PC3 flu cells. The slight difference in SERS intensity in the latter case between 10 pM and 20 pM can be attributed to the non-specific internalization of the SERS agent. More importantly, we observe that the SERS agent first appears at the cellular surface at low concentrations (10 pM and 20 pM), and only within the cell when the concentration of the SERS agents is higher (30 pM and 50 pM) in the PSMA+ PC3 PIP cells. These images suggest that the SERS agent can enter the PSMA+ PC3 PIP cell after targeting PSMA on the cell surface. Such an uptake process of the SERS agent is consistent with known internalization of corresponding low molecular weight agents.^39,40^ We further confirmed the internalization of the SERS agent through TEM (Fig. S7†). As more SERS agents home in on the PSMA+ PC3 PIP cell surface at higher concentrations, internalization of SERS agents increases thereby elevating spectral intensity levels and improving the imaging sensitivity.

## Conclusions

In summary, we have developed an optical platform for precise visualization of live prostate cancer cells based on vibrational spectroscopic imaging, tailored nanoprobes for plasmon enhancement and a small-molecule inhibitor of PSMA. The constructed agents provide significant signal enhancement and reproducible Raman spectral responses enabling quantitative measurement of biochemical species at low concentrations. SERS imaging is also complementary to and entirely compatible with other optical approaches such as dark field microscopy that can facilitate rapid characterization of agent loading at the sub-cellular level. We have demonstrated that the SERS imaging platform can identify a single prostate cancer cell owing to its high sensitivity and excellent specificity. These data support further preclinical feasibility studies, which are currently ongoing in our laboratories.

## Experimental section

### Chemicals and materials

Chloroauric acid (HAuCl_4_·*x*H_2_O, 99.999% trace metals basis), trisodium citrate dihydrate (HOC(COONa)(CH_2_COONa)_2_·2H_2_O, ≥99%), poly(vinylpyrrolidone) (PVP, (C_6_H_9_NO)_*n*_, molecular weight-10 kg mol^–1^), sodium borohydride (≥99%), *N*,*N*-dimethyformamide (DMF, anhydrous 99.8%), dimethyl sulfoxide (DMSO, 99.5%, molecular biology), sodium hydroxide (pellets, 99.99% trace metals basis), (3-aminopropyl) trimethoxysilane (APTMS, 97%), sodium silicate (Na_2_O(SiO_2_)*x*·*x*H_2_O, reagent grade), 4-nitrothiophenol (NTP, technical grade 80%), RPMI 1640, fetal bovine serum (FBS), penicillin-streptomycin and WST-1 assay reagents were purchased from Sigma-Aldrich (St. Louis, MO). Methoxy-poly(ethylene glycol)-silane (mPEG–silane, molecular weight – 2 kg mol^–1^) was obtained from Laysan Bio (Arab, AL). Phosphate buffered saline (1× PBS, pH 7.4) solution was purchased from Quality Biology (Gaithersburg, MD). PYREX® Petri Dishes were purchased from Corning Incorporated (Corning, NY), and quartz coverslips from Alfa Aesar (Ward Hill, MA). All other reagents and solvents used in this study were of analytical grade and used without further purification.

### Synthesis of SERS tags

NTP-encoded SERS tags were synthesized according to our previously reported procedure.^33,41^ Gold nanostars (GNS) were first synthesized by the seed-mediated growth method.^24^ Briefly, to prepare the gold seed solution, 1 mL of 1 wt% HAuCl_4_·*x*H_2_O aqueous solution was diluted to 90 mL with deionized water followed by the addition of 2 mL 38.8 mM trisodium citrate aqueous solution. 1 mL of freshly prepared NaBH_4_ solution (0.075 wt% in 38.8 mM trisodium citrate aqueous solution) was then slowly added. After the reaction was kept at room temperature overnight, 50 mL of the gold seed solution was mixed with PVP (10 mM) at room temperature followed by constant stirring for 24 h to obtain the PVP-coated gold seed solution. Next, 82 μL of 50 mM HAuCl_4_·*x*H_2_O aqueous solution was added to 15 mL DMF of 10 mM PVP, and then 86 μL of PVP-coated gold seed solution was rapidly added with constant stirring at room temperature for 3 h. The extinction spectrum shows no change when the growth time exceeds 30 min, indicating that the growth of GNS is completed within 30 min. The resulting GNS were successively centrifuged at 11 000 rpm for 15 min and washed at least three times with ethanol, and re-suspended into 15 mL deionized water for preparation of SERS tags. To prepare the SERS tag, a freshly prepared solution of NTP (10 μM) was added dropwise to the aforementioned GNS colloidal solution under continuous magnetic stirring. After 30 min, 10 μL of freshly prepared 50 mM APTMS ethanolic solution was added and stirring was continued for another 30 min. The pH value of reaction solution was adjusted to *ca.* 9–10 by addition of aq. NaOH solution. Following this, 200 μL of freshly prepared trisodium silicate solution (0.54 wt%) was added slowly, and then stirred for one day. 5 mL anhydrous ethanol was subsequently added to generate a condensed silica layer. The reaction solution was kept standing for one more day, then centrifuged and washed with anhydrous ethanol and deionized water, respectively. The pellets were re-dispersed into 1× PBS for further use.

### Synthesis of SERS agents

The prepared SERS tags were conjugated with a low-molecular weight, urea-based PSMA-targeting moiety, Glu-urea-Lys-linker-NHS, which we have previously reported.^5^ To improve biocompatibility, SERS tags were first co-modified by mPEG–silane and APTMS with a 1 : 2 molar ratio. Typically, 5.65 μM mPEG–silane and 11.3 μM APTMS were added to 50 pM SERS tag ethanolic solution. After the reaction continued for 12 h with magnetic stirring, the solution was centrifuged and washed with ethanol and deionized water, respectively. The resulting solids were re-dispersed into 1× PBS followed by dropwise addition of Glu-urea-Lys-linker-NHS (10% v/v) in DMSO within 30 min under a shaking rate of 60 rpm. Next, the solution was shaken at 60 rpm for 2 h. The reaction solution was washed at least 4 times with 1× PBS (4 °C) to remove excess unbound Glu-urea-Lys-linker-NHS, and the pellets from the final wash were the conjugates called SERS agents, and re-dispersed into 1 mL 1× PBS.

### Cell culture

Human PC3 prostate cancer cells engineered to over-express PSMA (PSMA+ PC3 PIP) as well as isogenic wild-type cells (PSMA- PC3 flu) were originally obtained from Dr Warren Heston (Cleveland Clinic, Cleveland, OH). Both PSMA+ PC3 PIP and PSMA- PC3 flu cell lines were incubated in RPMI 1640 supplemented with 10% FBS and 1% penicillin-streptomycin in a humidified incubator at 37 °C/5% CO_2_. For SERS imaging of live cells, the cells (1 × 10^6^ cells per mL) were seeded in a 60 mm quartz-bottomed Petri dish, and then allowed to adhere to the quartz-bottomed dish overnight. SERS agents of various concentrations (10, 20, 30 and 50 pM) were added to the Petri dish, and incubated for 1 h followed by replacement of incubation medium with freshly prepared RPMI 1640 incubation medium and was kept standing for 12 h before SERS imaging was performed.

### *In vitro* cellular cytotoxicity test

The cytotoxicity of the SERS agents was evaluated by a WST-1 assay. Cells (1 × 10^4^ cells per well) were seeded onto 96-well plates and incubated for 24 h in RPMI 1640 supplemented with 10% FBS and 1% penicillin-streptomycin. Then, 50 pM SERS agents was added, and after incubation for a pre-set time, the WST-1 reagent (4-[3-(4-lodophenyl)-2-(4-nitrophenyl)-2*H*-5-tetrazolio]-1,3-benzene disulfonate) was added into the corresponding wells and incubated for 1 h in the 37 °C/5% CO_2_ incubator before the absorbance was measured.

### SERS measurements

For SERS measurements, an inverted confocal Raman microscope was built by adopting a similar design to that described in our previous publication.^33^ A compact LM series volume holographic grating-stabilized laser diode (*λ*_em_ = 785 nm) (Ondax) with a clean-up filter (LL01-785-12.5, Semrock) was used as the excitation source and re-directed to the dual-axes galvanometer mirrors (GVS112, Thorlabs). The use of the galvanometer mirrors enables high speed XY scanning in the sample plane. A 0.65–1.25 NA, 60× oil immersion objective lens (RMS60X-PFOD, Olympus) was used to focus the laser beam on and collect the Raman scattered light from the sample. The backscattered light was collected by a 50 μm multimode fiber (M14L01, Thorlabs), delivered to an HoloSpec f/1.8 spectrograph (Kaiser Optical Systems, Andor) and the dispersed light was finally detected by an iDus CCD Camera (DU420A-BEX2-DD, Andor). Customized LabView 2013 (National Instruments) and MATLAB 2013 (Mathworks) modules were used to control the system, acquire the data, and analyze the data. Raman and SERS spectra were recorded using 5 mW laser power and 1 s integration time, unless otherwise mentioned.

### Characterization

Extinction spectra for the GNS and SERS tags were recorded on a Shimadzu UV-2401 spectrometer. Transmission electron micrographs were acquired using the FEI Tecnai G2 Spirit TWIN transmission electron microscope (TEM) at an accelerating voltage of 120 kV. The samples for TEM were prepared by deposition of a drop of the suspensions in ethanol onto ultrathin Formvar-coated 200 mesh copper grids (Ted Pella, Inc.) and left to dry in air. Cell samples for TEM imaging were prepared according to the procedure reported in the literature,^42^ after incubation as described above. Briefly, after incubation with the SERS agents, cells were fixed in a 0.1 M sodium cacodylate buffer solution (pH 7.4) containing 3.0% formaldehyde, 1.5% glutaraldehyde, 5.0 mM CaCl_2_ and 2.5% sucrose for 1 h, rinsed with 0.1 M sodium cacodylate buffer solution (pH 7.4) of 2.5% sucrose, and then post-fixed for 1 h in Palade's buffered solution of 1% osmium tetroxide. After dehydration with graded series of cold ethanol (70, 90 and 100%), the samples were washed three times with fresh 100% ethanol, and then twice with propylene oxide at room temperature before being transferred to fresh 100% Epon and embedded in fresh 100% Epon under vacuum for 4–6 h. After being kept at 60 °C for 24–48 h, serial sections were cut and mounted onto copper-grids for examination by TEM.
