# Supplementary material for: Ultrahigh affinity Raman probe for targeted live cell imaging of prostate cancer
Source: Chem Sci. 2016 Jul 15;7(11):6779–85. doi: 10.1039/c6sc01739h (PMC5356002; doi:10.1039/c6sc01739h)
Supplement: Supplementary file 1 [file SC-007-C6SC01739H-s001.pdf]

Electronic Supplementary Material (ESI) for Chemical Science.
This journal is © The Royal Society of Chemistry 2016

Supplementary Information

Ultrahigh Affinity Raman Probe for Targeted Live Cell Imaging of Prostate Cancer

Ming Li,*[a] Sangeeta Ray Banerjee,[b,c] Chao Zheng,[a,d] Martin G. Pomper,*[b,c] and Ishan Barman*[a,b]

[a]Department of Mechanical Engineering, Johns Hopkins University, Baltimore, Maryland 21218, United States

[b] The Sidney Kimmel Comprehensive Cancer Center, Johns Hopkins University School of Medicine Baltimore, Maryland 21287, United States

[c]The Russell H. Morgan Department of Radiology and Radiological Sciences, Johns Hopkins University

[d]Department of Breast Surgery, the Second Hospital of Shandong University, Jinan, Shandong 25000, China

*To whom the correspondence should be addressed.

E-mails: liming0823@gmail.com (M. L.), ibarman@jhu.edu (I.B.) and mpomper@jhmi.edu (M.G.P.)

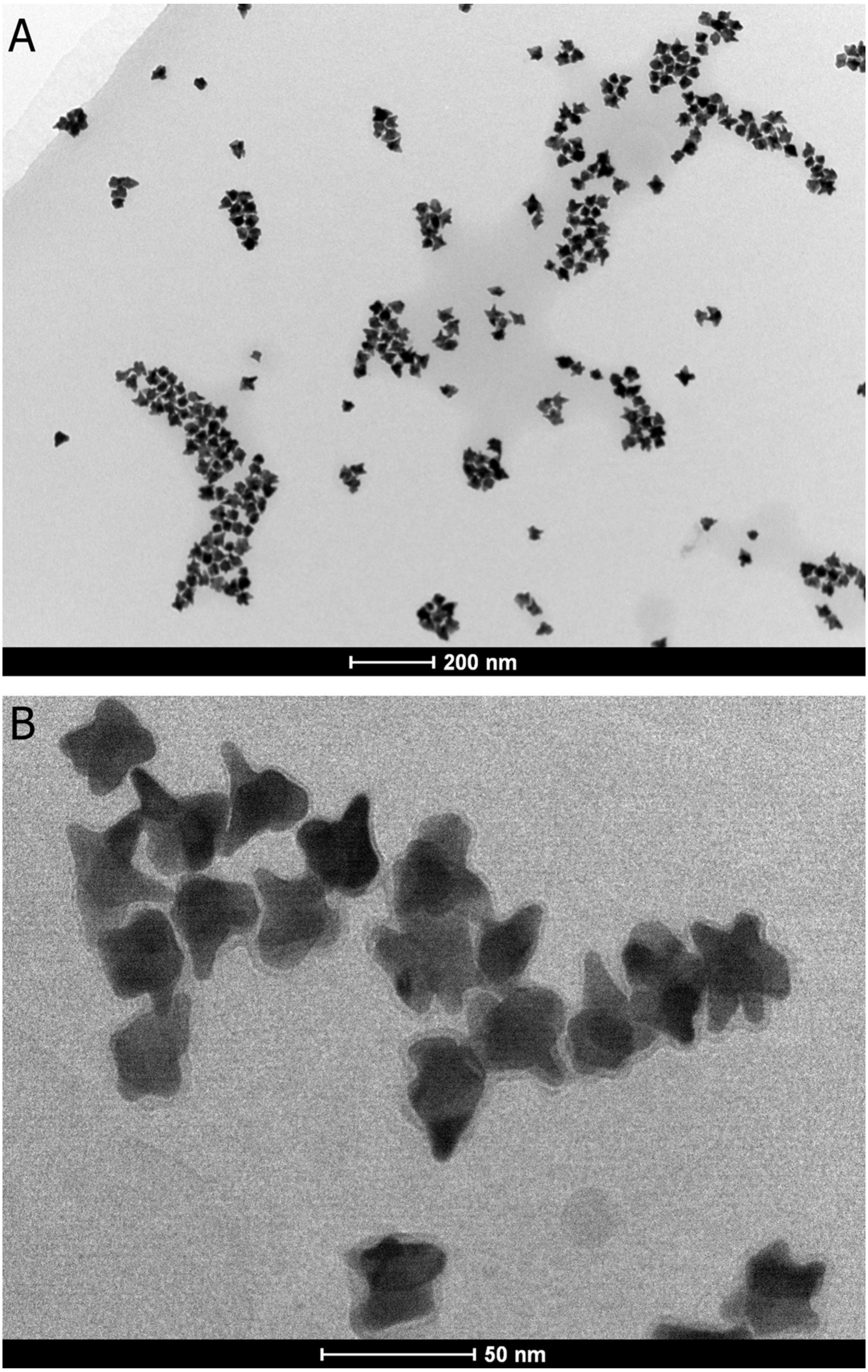

Figure S1. TEM images of (A) synthesized gold nanostars and (B) SERS tags.

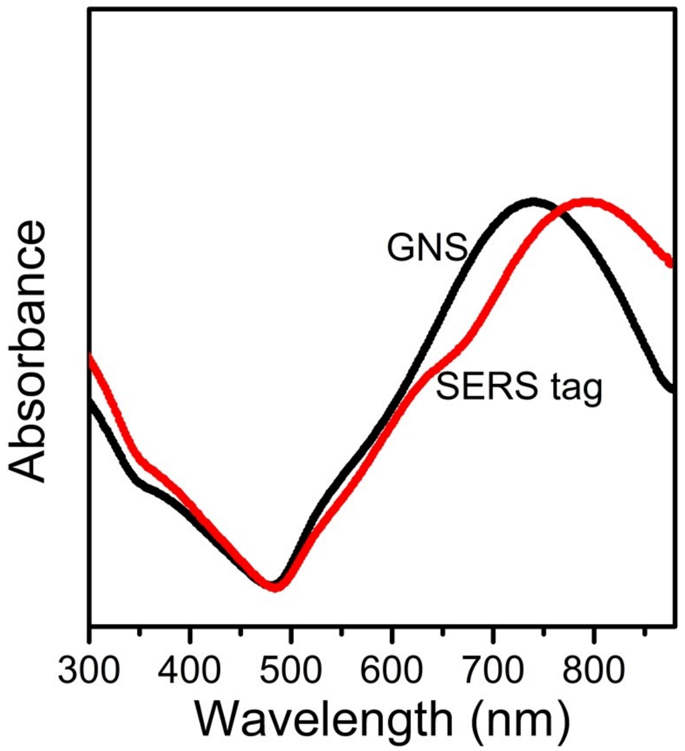

Figure S2. Extinction spectra of gold nanostar (GNS) and constructed SERS tag. The SERS tag was prepared by sandwiching Raman reporter (NTP) molecules between the GNS and the thin outer silica shell. The localized surface plasmon resonance (LSPR) absorbance maximum of GNS is 750 nm, and the LSPR absorbance maximum of SERS tag is 780 nm. The 30 nm red-shift of the LSPR maximum is ascribed to the change in refractive index because of the silica coating.

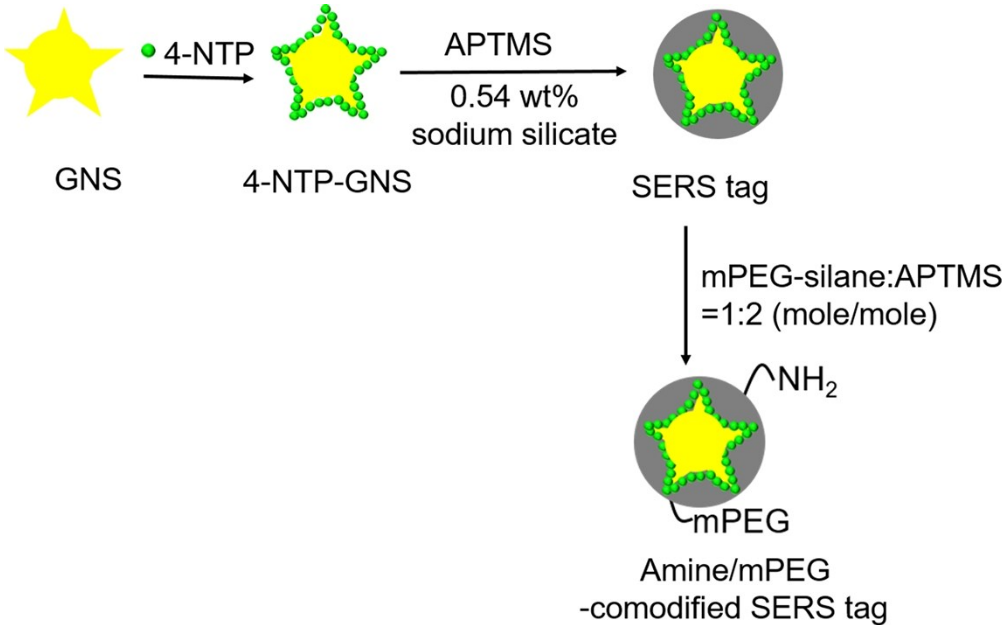

Figure S3. Schematic illustration of SERS tag and its co-modification with amine and mPEG terminal groups. NTP molecules were chemically adsorbed onto the GNS surface by the –S-Au interaction, followed by coating with a 3-4 nm silica layer. PEGylation was performed to improve biocompatibility, and amine modification was performed to permit conjugation with the urea-based small molecule PSMA targeting moiety.

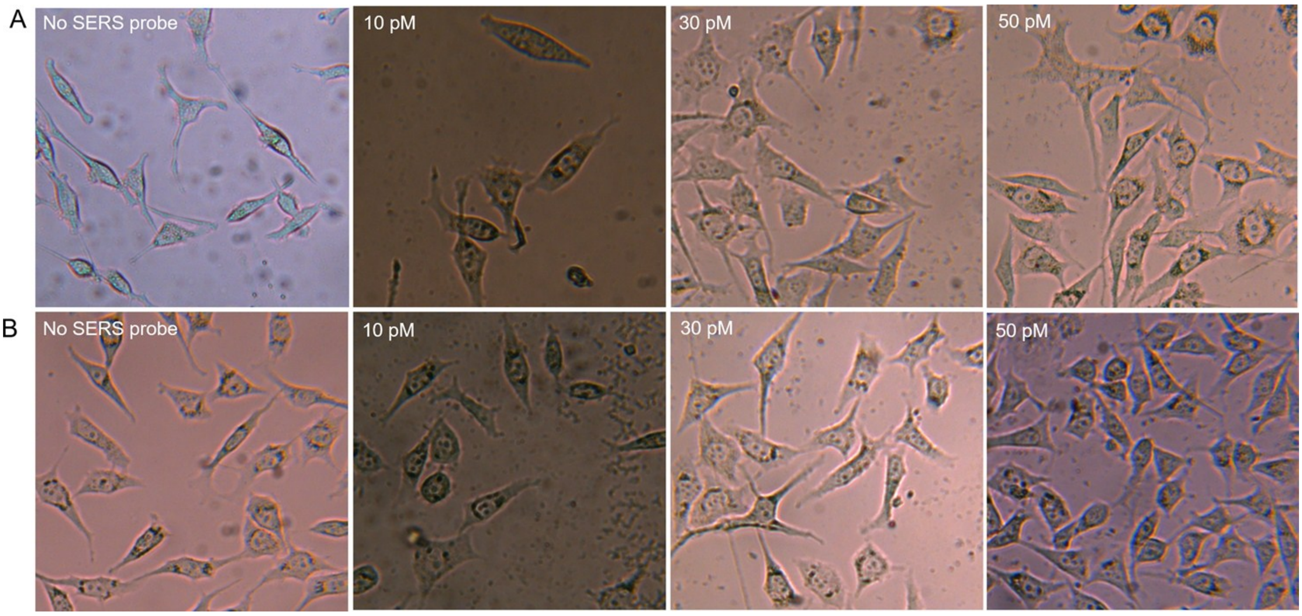

Figure S4. Bright-field images of (A) PSMA+ PC3 PIP cells and (B) PSMA- PC3 flu cells before and after incubation with SERS agents of representative concentrations (10, 30 and 50 pM). The images show that cells adhere to the quartz-bottomed petri dish both in the absence of SERS agents as well when incubated in the presence of various doses of SERS agents. Expectedly, no significant difference is observed between PSMA+ PC3 PIP cells and PSMA- PC3 flu cells, both in presence and absence of the SERS agents.

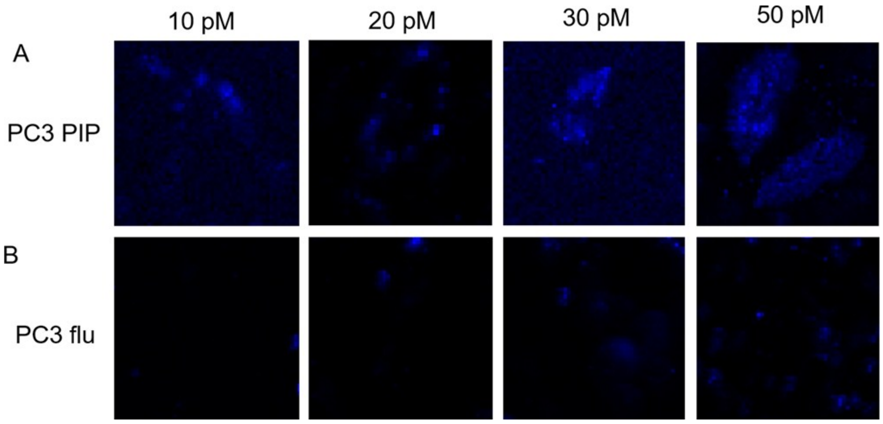

Figure S5. SERS images of PSMA+ PC3 PIP cells and PSMA- PC3 flu cells after incubation in various doses of SERS agents (10, 20, 30 and 50 pM). The SERS images were constructed on the basis of the integrated intensity of the 1340 cm^{-1} Raman band.

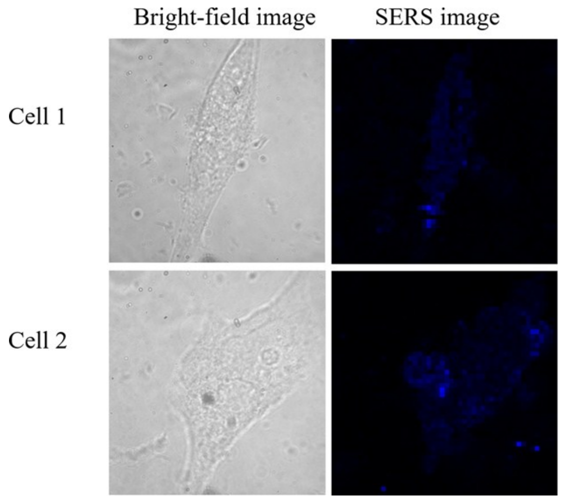

Figure S6. Two additional bright-field and SERS images of PSMA+ PC3 PIP cells after incubation with 50 pM of SERS agents. These images confirm the reproducibility of targeted SERS imaging of PSMA+ PC3 PIP cells. The SERS images were constructed on the basis of the integrated intensity of the 1340 cm^{-1} Raman band.

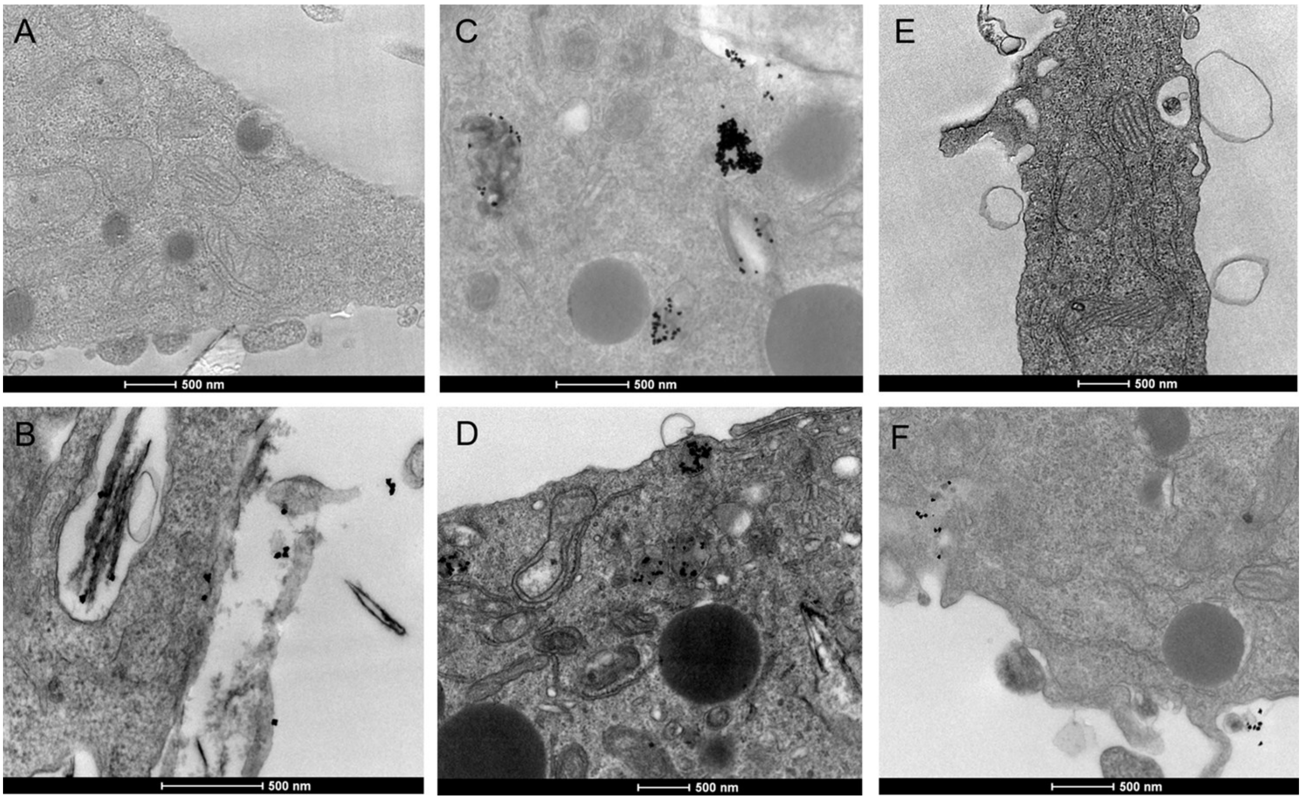

Figure S7. Transmission electron microscopy (TEM) images of PSMA+ PC3 PIP cells (A) in the absence of SERS agents, and in the presence of (B) 10 pM and (C, D) 50 pM SERS agents. (E,F) TEM images of PSMA- PC3 flu cells in the absence and presence of 50 pM SERS agents, respectively. Evidently, more SERS agents are internalized in the PSMA+ PC3 PIP cells than the PSMA- PC3 flu cells. The SERS agents initially home in around the PSMA expressed at the cell surface; but as higher concentrations of SERS agents are used, some SERS agents are internalized into the cells through the assistance of PSMA and can be found to reside within endosomes. Significantly lesser number of SERS agents enter the PSMA- PC3 flu cells even at higher concentrations.
